# Case Report: Opportunities for Treatment of Severe COVID-19 Patients—Lessons From a Death Case

**DOI:** 10.3389/fmed.2020.00533

**Published:** 2020-08-14

**Authors:** Yan Wang, Zhuo Niu, Jia-Lin Peng, Hui-Sheng Wang, Ke Zhang

**Affiliations:** ^1^Department of Respiratory Medicine, The People's Hospital of Liaoning Province, Shenyang, China; ^2^Key Laboratory of Cell Biology, Ministry of Public Health, Key Laboratory of Medical Cell Biology, Ministry of Education, Department of Developmental Cell Biology, China Medical University, Shenyang, China; ^3^Key Laboratory of Precision Diagnosis and Treatment of Gastrointestinal Tumors, Ministry of Education, Department of Surgical Oncology and General Surgery, The First Affiliated Hospital of China Medical University, Shenyang, China; ^4^Department of Neurology, Nan Zhang People's Hospital, Xiangyang, China; ^5^Department of Medical Service, The People's Hospital of Liaoning Province, Shenyang, China

**Keywords:** SARS-CoV-2, COVID-19, severe, rescue, treatment

## Abstract

With the spread of severe acute respiratory syndrome coronavirus 2 (SARS-CoV-2) infection, the incidence of coronavirus disease (COVID-19) increases each day. To date, there is no specific anti-SARS-CoV-2 drug. The usual approach to treating COVID-19 is treating its symptoms. However, this approach is limited by the different conditions of each area. We treated a 57-year-old man who was initially diagnosed with a severe type of the infection, but he progressed to a critical condition and eventually died. We learned valuable lessons from this case. The first lesson is the need to use immediate invasive mechanical ventilation if there is no obvious improvement after using non-invasive ventilation for several hours, which directly affects the prognosis. Another lesson is the risk involved in transferring severe COVID-19 patients. In the process of transfer, various threats may be encountered at any time. Thus, accurate assessment of the patient's condition and strict medical conditions are highly required. During the patient's 25-day treatment, we performed cardiopulmonary resuscitation twice. Currently, many patients require invasive mechanical ventilation and transfer to a superior hospital. We hope our findings will provide some advice and help for treating severe and critical COVID-19 cases.

## Introduction

In December 2019, a novel coronavirus, namely, severe acute respiratory syndrome coronavirus 2 (SARS-CoV-2), was discovered in several similar pneumonia cases in Wuhan, China (1, 2). The disease caused by SARS-CoV-2 was officially named coronavirus disease (COVID-19) (3). The appearance of SARS-CoV-2 resulted in a pandemic due to the population's high susceptibility to the virus, which is mainly transmitted via respiratory droplets, contact, and other potential routes, such as fecal-oral transmission and aerosols (4).

According to the latest Diagnosis and Treatment Guidelines published by the Ministry of Health of China (4), the clinical presentation of COVID-19 is classified as mild, moderate, severe, and critical. Patients presenting with respiratory distress (respiratory rate [RR] ≥30 breaths/min), low oxygen saturation at rest (SpO_2_ ≤ 93%), or arterial partial pressure of oxygen (PaO_2_)/fraction of inspired oxygen (FiO_2_) ≤ 300 mmHg are diagnosed as severe cases; critical cases develop worse conditions, such as respiratory failure requiring mechanical ventilation, shock, or other organ failure, requiring an intensive care unit.

There is no specific COVID-19 drug so far, and the approach to treating the infection is usually based on treating symptoms, including respiratory support, antiviral medication, and antibiotics. However, there are difficulties to this approach due to limiting conditions in different areas. Here, we report a case of a patient who was initially diagnosed as a severe case, but he progressed to a critical case and eventually died. The lessons we learned will be helpful for rescuing severe cases of COVID-19 under the current epidemic situation.

## Case Description

A 57-year-old man (height: 177 centimeters, weight: 86 kilograms) presented with a fever of 38.5°C for 4 days accompanied by fatigue and dyspnea, and he visited the clinic on February 4, 2020. He had an 8-year history of hypertension without other diseases. He disclosed a history of contact with a confirmed case.

**Figure 1:**
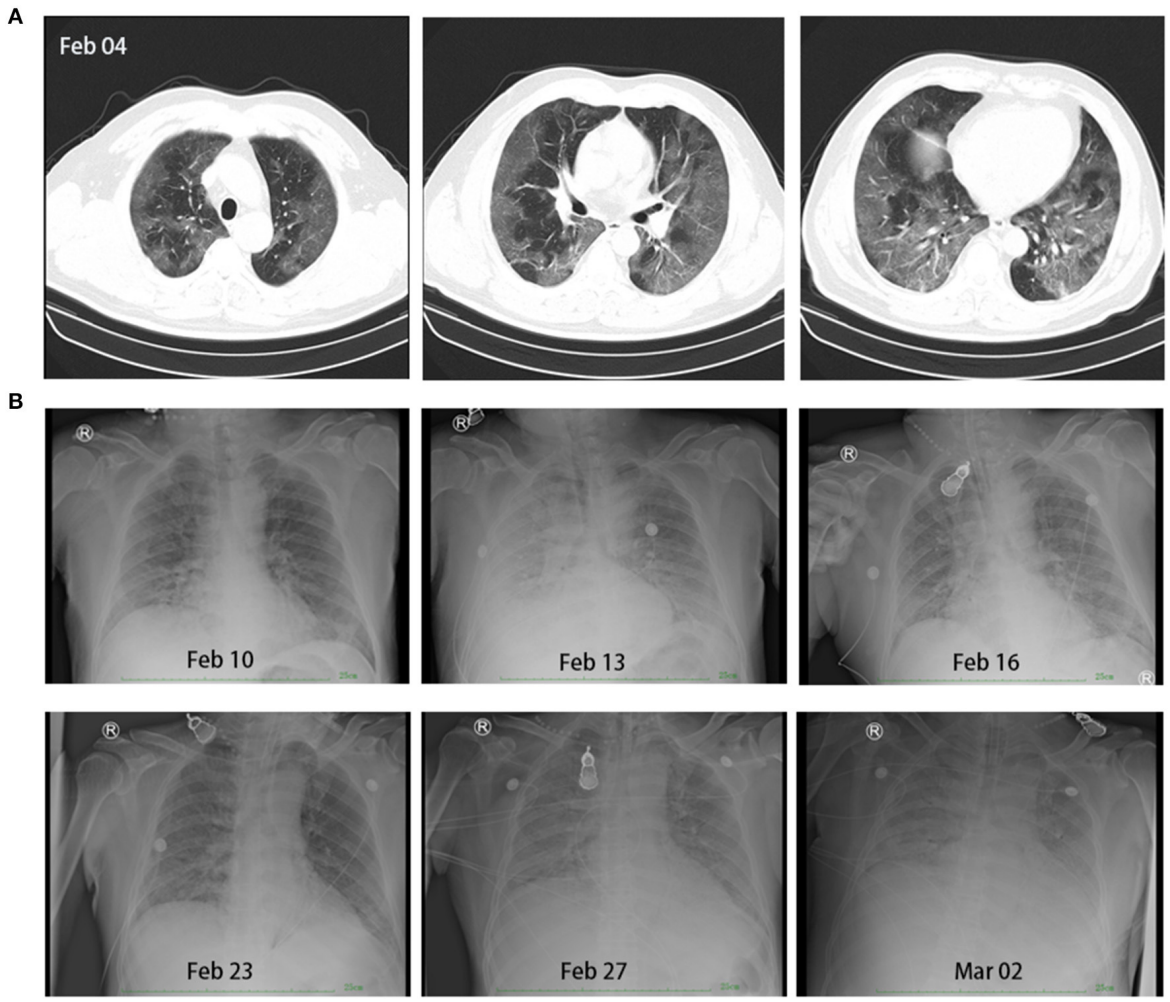
Chest-computed tomography of the patient on February 4, 2020, showed signs of infection indicating the possibility of viral pneumonia **(A)**. Continuous chest digital radiography during treatment from February 10 to March 2 **(B)**.

**Figure 2:**
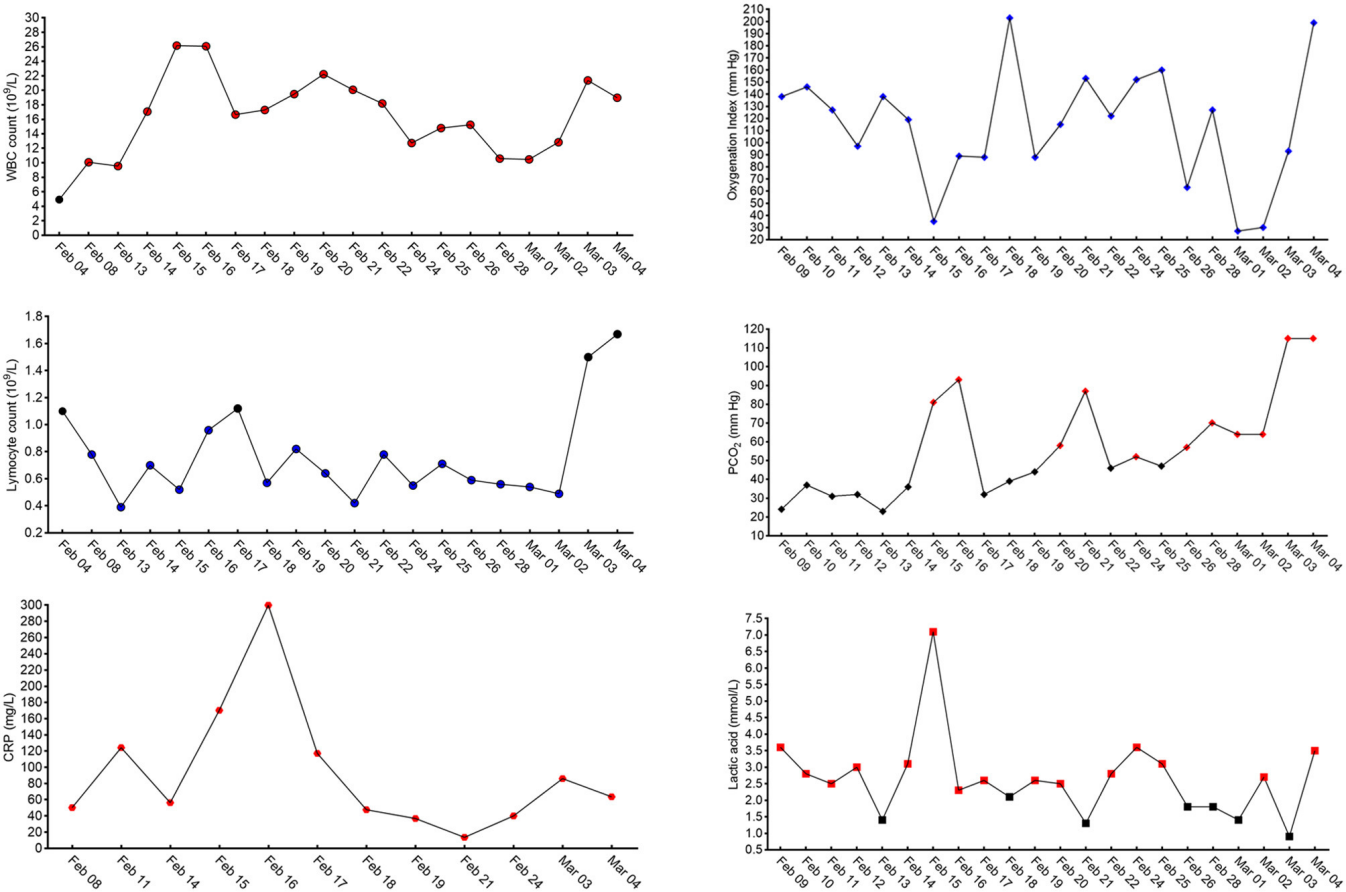
Continuous analysis of blood cells and blood gas during treatment: normal white blood cell count, 4.0–10.0 × 10^9^ cells/L; normal lymphocytes, 1.1–3.2. × 10^9^ cells/L; normal C-reactive protein, <5 mg/L; normal oxygenation index (>400 mmHg); normal PCO_2_ (<50 mmHg); and normal lactic acid, 0.2–1.4 mmol/L. The black symbols indicate the result is in the normal range, the red symbols indicate the results are above the normal range, and the blue symbols indicate the results are below the normal range.

As he was febrile, the screening test for COVID-19 was performed immediately. Chest-computed tomography (CT) showed diffuse ground-glass opacities in the periphery of bilateral lungs, which represented the characteristic imaging for pneumonia of COVID-19 (Figure 1). Blood tests revealed that the white blood cell count was 4.92 × 10^9^ cells/L (normal: 4.0–10.0 × 10^9^ cells/L), lymphocyte (LY) was 1.1 × 10^9^ cells/L (normal: 1.1–3.2 × 10^9^ cells/L), C-reactive protein (CRP) was 118.24 mg/L (normal: <5 mg/L), and pathogenic findings of common respiratory viruses and influenza A and B viruses were negative (Figure 2). He was diagnosed with suspected COVID-19.

On admission, he had a body temperature of 38.8°C, blood pressure (BP) of 140/80 mmHg, RR of 20 breaths/min, and a heart rate (HR) of 98 beats/min. SpO_2_ was 84% without oxygen supply. He was supported with high flow oxygen (50 L/min, FiO_2_ 60%) and treated with antimicrobial and antiviral drugs, including levofloxacin (oral, early stage), moxifloxacin (intravenous, later), and ribavirin. Considering the changes noted on chest CT, the patient was diagnosed with acute respiratory distress syndrome (ARDS), and methylprednisolone (40–160 mg/day intravenously from February 4 to February 9) and gamma-globulin (10 g/day intravenously from February 5 to February 10) were administered. On February 7, the Centers for Disease Control confirmed that the patient's oropharyngeal swab test for SARS-CoV-2 was positive; thus, the patient was diagnosed with COVID-19 (severe case), ARDS, and hypertension. During the treatment course, the patient's body temperature returned to normal. Meanwhile, PaO_2_ improved gradually and CRP levels decreased to 50.3 mg/L on February 9. However, the patient's respiratory distress and fatigue did not relieve, and CRP levels increased to 124.2 mg/L on February 11 (Figure 2).

On February 12, PaO_2_ fell to 58 mmHg. Non-invasive mechanical ventilation was performed, and another course of methylprednisolone was administered. We suggested a change to invasive mechanical ventilation because there was no improvement in respiratory distress after 2 h, but his family refused. PaO_2_ and CRP seemed to improve with increasing FiO_2_ (up to 100%) and the use of methylprednisolone, but the clinical manifestation persisted, and, on February 15, PaO_2_ could not be maintained; it fell to 35 mmHg, the SpO_2_ was at 45%, the RR at 50 breaths/min, and confusion occurred. Invasive mechanical ventilation was authorized, but ventricular fibrillation occurred after endotracheal intubation. Cardiopulmonary resuscitation (CPR) and defibrillation were performed immediately, during which no blood pressure and heartbeat were detected for 12 min. The patient recovered but was in a deep coma. We adjusted the ventilator settings to a pressure synchronized intermittent mandatory ventilation (P-SIMV) mode with a maximum airway pressure (P_max_) of 18 cmH_2_O, positive end expiratory pressure (PEEP) of 15 cmH_2_O, FiO_2_ of 100%, and frequency of 18 breaths/min. We changed to a superior antibiotic after blood test results showed leukocytosis (26.17 × 10^9^ cells/L), lymphopenia (0.52 × 10^9^ cells/L), and elevated CRP levels (170.2 mg/L) (Figure 2). However, on February 17, the urine volume was 1,100 mL, and renal failure occurred, with elevated blood urea nitrogen (25.24 mmol/L, normal: 3.2–7.1. mmol/L) and creatinine (395.2 μmol/L, normal: 44.0–133.0 μmol/L) levels (Figure 2). We adjusted the ventilator to pressure-assist/control mode with PEEP of 11 cmH_2_O and FiO_2_ of 100%, and we operated continuous renal replacement therapy (CRRT) once. On February 18, renal function improved, and the urine volume reached 4,400 mL. Over this period, the patient progressed to a critical case, and bedside continuous chest digital radiography (DR) was used for monitoring. We observed faded imaging and restoration of organic function after treatment (Figure 1).

On February 24, the patient's condition remained stable and the ventilator setting was changed to P-SIMV mode. The HR was 90 beats/min, BP was 132/78 mmHg, RR was 20 breaths/min, and SpO_2_ was 96% (FiO_2_ was 60%). Considering the policy of centralized management for better treatment, the patient was to be transferred to a superior hospital on February 25. We prepared an ambulance equipped with a limited ventilator, which was a PCV model with a P_max_ of 25 cm H_2_O and FiO_2_ of 100%, but it did not offer the possibility to apply PEEP and a critical care specialist; we also estimated and prepared for other potential risks. However, after 10 min in the ambulance, the patient's SpO_2_ suddenly fell to 0%, the HR slowed down to 20 beats/min, and he also presented with severe cyanosis and respiratory distress. CPR was performed, and the ambulance returned immediately. A specialized ventilator was used again with P_max_ of 30 cmH_2_O, PEEP of 15 cmH_2_O, and FiO_2_ of 100%. Although the chest DR showed progress on February 27 (Figure 1), the patient's condition worsened, and he died on March 4.

## Discussion

Since the beginning of the global COVID-19 pandemic, clinicians and scientists have worked hard to ensure the recovery of patients. Now, it is crucial to grasp every opportunity to rescue severe cases and reduce mortality.

In this case, the characteristic CT imaging changes and positive pathological test helped us diagnose COVID-19 (severe case) with SpO_2_ of 84% on admission. During the initial treatment, symptomatic treatment was administered, and the patient's body temperature returned to normal. High-flow oxygen support was also applied. However, the clinical manifestation of respiratory distress did not relieve significantly because of a persistently low PaO_2_/FiO_2_ (≤ 300 mmHg). On February 12, PaO_2_ fell to 58 mmHg, and non-invasive mechanical ventilation was attempted (5). Nonetheless, no obvious improvement was observed after 2 h. Under these circumstances, invasive mechanical ventilation was proposed according to the guidelines; however, the patient's family refused. Notably, the patient remained awake when blood gas analysis indicated severe hypoxia. This mismatch between clinical manifestation and objective medical indication misled the subjective judgment of his family on his condition. This phenomenon is usually not observed in ARDS caused by other pathogens, and its cause remains unknown.

Although PaO_2_ seemed to improve on February 13 and 14 when supported with pure oxygen, it became worse on February 15. Invasive mechanical ventilation was performed as a salvage treatment, and the patient developed ventricular fibrillation, renal failure, and left brain damage with a long-term deep coma state. Thus, the first lesson we learned is that we missed the best timing for invasive mechanical ventilation due to the discrepancy between the clinical picture and the ongoing severe oxygenation failure, the objections of the family, and the sudden aggravation. This constellation caused the patient to remain at high risk, increased the difficulty of subsequent treatment, and influenced the prognosis negatively. Here, we highlighted the latest guideline published by the Ministry of Health of China (the 7th edition), where oxygen supply was a major symptomatic treatment, beginning with a nasal catheter or face mask, and, if there is no relief, high-flow oxygen supply or non-invasive mechanical ventilation should be considered (6). Moreover, after no relief for just a short time (1–2 h), we suggested initiate invasive mechanical ventilation in a timely manner (7).

China has introduced the following principles for the treatment of COVID-19: centralized patients, centralized experts, centralized resources, and centralized rescue (8). These principles have rescued many patients, even the ones that needed invasive mechanical ventilation for a long time. As for this case, the patient was only 57-years-old with no severe preexisting diseases that affected his health and prognosis. Therefore, given the following conditions of long-time mechanical ventilation, which cannot be removed, the parameter of mechanical ventilation remaining high with high pressure and inspired oxygen concentration, and a respiratory status showing signs of improvement, indicating an ability to maintain stable vital signs, we considered the transfer to a superior hospital for further treatment.

However, the patient's SpO_2_ fell and HR slowed down during the transfer, which made us return. Chest DR showed progress on February 27, and organ failure developed later. Eventually, regardless of our treatment, the patient died on March 4. Here, we seriously consider that the vehicle-borne simple ventilator we prepared was insufficient to maintain the pressure and volume of the airway, which resulted in severe impairment of oxygenation and the falling of SpO_2_ during the transfer. This is the second lesson we learned.

During the 25 days of treatment, the patient experienced CPR twice. Here, we draw two conclusions from this death case. First, invasive mechanical ventilation should be applied as early as possible when it meets the standard. A relatively strong resistance to hypoxia may misguide this judgement. Second, the patient's status and transfer conditions must be assessed before transfer. A simple ventilator may not be suitable for transfer, and a specialized ventilator may be needed. Currently, many patients require invasive mechanical ventilation and transfer to a superior hospital. We wish our lessons may provide some advice and help for treatment of severe and critical cases of COVID-19.

## Data Availability Statement

All datasets generated for this study are included in the article/supplementary material.

## Ethics Statement

Written informed consent was obtained from the individual(s) for the publication of any potentially identifiable images or data included in this article. The next-of-kin of the patient consent for the report of the case.

## Author Contributions

YW and KZ designed this work. YW, J-LP, and H-SW collected the data. ZN and KZ wrote and corrected the manuscript. All authors contributed to the article and approved the submitted version.

## Conflict of Interest

The authors declare that the research was conducted in the absence of any commercial or financial relationships that could be construed as a potential conflict of interest.
